# Berberine Attenuates Hyperglycemia by Inhibiting the Hepatic Glucagon Pathway in Diabetic Mice

**DOI:** 10.1155/2020/6210526

**Published:** 2020-01-02

**Authors:** Ying Zhong, Jing Jin, Peiyu Liu, Yu Song, Hui Zhang, Liang Sheng, Huifang Zhou, Bijie Jiang

**Affiliations:** ^1^Department of Pharmacology, School of Basic Medical Science, Nanjing Medical University, 101 Longmian Rd, Nanjing, Jiangsu 211166, China; ^2^Department of Gynecology, Affiliated Hospital of Nanjing University of Chinese Medicine, Nanjing, Jiangsu 210029, China; ^3^Pharmacy College, Xinxiang Medical University, 601 Jinsui Avenue, Xinxiang, Henan 453003, China; ^4^Key Laboratory of Rare Metabolic Diseases, Nanjing Medical University, 101 Longmian Rd, Nanjing, Jiangsu 211166, China; ^5^School of Public Health, Xinxiang Medical University, 601 Jinsui Avenue, Xinxiang, Henan 453003, China

## Abstract

Dysregulated glucagon drives hyperfunction in hepatic glucose output, which is the main cause of persistent hyperglycemia in type 2 diabetes. Berberine (Zhang et al., 2010) has been used as a hypoglycemic agent, yet the mechanism by which BBR inhibits hepatic gluconeogenesis remains incompletely understood. In this study, we treated diabetic mice with BBR, tested blood glucose levels, and then performed insulin, glucose lactate, and glucagon tolerance tests. Intracellular cAMP levels in hepatocytes were determined by ELISA, hepatic gluconeogenetic genes were assayed by RT-qPCR, and the phosphorylation of CREB, which is the transcriptional factor controlling the expression of gluconeogenetic genes, was detected by western blot. BBR reduced blood glucose levels, improved insulin and glucose tolerance, and suppressed lactate- and glucagon-induced hepatic gluconeogenesis in ob/ob and STZ-induced diabetic mice. Importantly, BBR blunted glucagon-induced glucose production and gluconeogenic gene expression in hepatocytes, presumably through reducing cAMP, which resulted in the phosphorylation of CREB. By utilizing a cAMP analogue, adenylate cyclase (AC), to activate cAMP synthetase, and an inhibitor of the cAMP degradative enzyme, phosphodiesterase (PDE), we revealed that BBR accelerates intracellular cAMP degradation. BBR reduces the intracellular cAMP level by activating PDE, thus blocking activation of downstream CREB and eventually downregulating gluconeogenic genes to restrain hepatic glucose production.

## 1. Introduction

Berberine (BBR), an isoquinoline-type alkaloid originally isolated from *Coptis chinensis* with a long history of Chinese medicinal application, has been shown to reduce blood glucose levels in diabetes [1, 2]. Hyperglycemia of diabetic patients is largely caused by sthenic glucose production in the liver [3]. The hypoglycemic effect of BBR is due to its inhibition of hepatic gluconeogenesis [4, 5].

Previous views that BBR downregulates hepatic gluconeogenesis via activation of adenosine monophosphate-activated protein kinase (AMPK) have been challenged by recent investigations that confirmed that AMPK is not necessary or at least not essential for BBR to regulate hepatic gluconeogenesis [6–9]. Thus, how berberine downregulates gluconeogenesis remains unclear.

Hepatic gluconeogenesis is physiologically initiated by glucagon, which activates adenylyl cyclase (AC) to increase the cytosol cyclic AMP (cAMP) level via its receptor on the hepatocyte plasma membrane. cAMP stimulates PKA to phosphorylate cyclic AMP response element binding (CREB), a transcriptional factor that regulates gluconeogenetic genes such as phosphoenolpyruvate carboxykinase (Pepck) and glucose-6-phosphatase (G6pc), and thus increases gluconeogenesis flux [10–12]. An abnormally elevated glucagon level and increased hepatic glucagon sensitivity are the primary reasons for hyperglycemia in type 2 diabetic patients [13, 14]. Therefore, a target that is commonly used for diabetic therapy is the glucagon signaling pathway in hepatocytes.

In the present study, it was confirmed that BBR targets the glucagon signaling pathway. BBR decreases glucagon-stimulated cAMP levels by activating phosphodiesterase (PDE), the catabolic enzyme of cAMP, which then inhibits hepatic gluconeogenesis. These molecular mechanisms by which BBR operates might provide new strategies to prevent diabetes and related metabolic complications.

## 2. Materials and Methods

### 2.1. Animal Experimental Procedures and Materials

All mice were maintained in a temperature-controlled (22 ± 2°C) environment with a 12 h light/dark cycle with free access to standard laboratory chow and water. The animal husbandry and experimental procedures complied with the guidelines of the Animal Care and Ethical Committee of Nanjing Medical University.

The ob/ob mice were purchased at the age of 15 weeks from the Animal Core Facility of Nanjing Medical University. According to procedures from previous reports [15, 16], the mice were randomized to two groups to receive berberine (BBR, 5 mg/kg/day, Sigma-Aldrich, St. Louis, MO) or saline (control) intraperitoneally while consuming a normal chow diet. Following a 3-week treatment, the mice were subjected to analysis of blood glucose levels (fasted and fed) and insulin levels in plasma, insulin tolerance test (ITT), glucose tolerance test (GTT), and glucagon (Sigma) and lactate (Sigma) tolerance tests.

Male C57Bl/6J mice at the age of 10 weeks were purchased from the Animal Core Facility of Nanjing Medical University. According to protocols from previous reports [17–19], mice were fasted overnight and intravenously injected via the tail with 100 mg/kg streptozotocin (STZ) (Sigma) dissolved in saline. One week after STZ injection, the diabetic mice were randomly divided into two groups: saline-treated (STZ) and BBR (5 mg/kg/day)-treated intraperitoneally while consuming a normal chow diet. Age-matched mice without STZ treatment receiving saline served as the normal group. After a 3-week treatment, the mice were subjected to analysis of blood glucose levels in fasted states and a GTT.

### 2.2. *In Vivo* Metabolic Assays

For the GTT and lactate tolerance test, the mice were fasted for 16 h, and then, 0.5 g/kg of glucose or lactate was administered by intraperitoneal injection. For the ITT or glucagon tolerance test, the mice were injected intraperitoneally with 4 U/kg of insulin (Humulin-R; Eli Lilly, Indianapolis, IN) or intravenously with 6 *μ*g/kg of glucagon after fasting for 5 or 15 h, respectively. Blood glucose was measured using an Xceed glucometer (Abbott Diabetes Care, UK), and plasma insulin was measured by an enzyme-linked immunosorbent assay (ELISA) (Millipore, Temecula, CA).

### 2.3. Primary Hepatocyte Culture

Primary hepatocytes were isolated from male C57Bl/6J mice using a collagenase perfusion as reported previously [20]. The cells were cultured in Williams' E medium (Sigma) supplemented with 6% fetal bovine serum (FBS) (Lonsa, Richmond, VA) and 1% penicillin/streptomycin (Hyclone, Utah) at 37°C in a humidified incubator under an atmosphere of 5% CO_2_-95% air.

### 2.4. Glucose Production in Hepatocytes

Once the hepatocytes were attached, the medium was switched to serum-free Williams' E medium for further culture for 5 hours. Then, cells were cultured in serum-free/glucose-free/phenol red-free Dulbecco's modified Eagle's medium supplemented with 15 mM lactate while being treated with glucagon (50 nM, Sigma) or BBR (10 *μ*M). Four hours later, the medium was used for the glucose assay by employing a Glucose Assay Kit (Jiancheng, Nanjing, China). Hepatocytes were lysed for the protein assay, which was performed using a Bradford protein assay kit (Jiancheng). The glucose production was calculated as the amount of glucose in medium normalized by the amount of protein in one hour.

### 2.5. cAMP Measurement

cAMP levels in hepatocytes and the liver were analyzed using a colorimetric cAMP ELISA kit (Cell Biolabs, San Diego, CA), according to the manufacturer's instructions. Briefly, hepatocytes in 12-well plates were incubated in serum-free Williams' E medium for 4 h and subjected to 10 *μ*M BBR for 15 min, followed by 50 nM glucagon for 10 min. Hepatocytes were lysed by 0.1 M HCl. cAMP concentrations were normalized to total cellular protein. ob/ob mice were fasted and treated with saline or 5 mg/kg body weight of BBR. One hour later, mice were injected intravenously with 15 *μ*g/kg glucagon, and livers were collected 15 min later.

### 2.6. Western Blotting

Extracts of the liver and hepatocytes prepared by RIPA buffer were separated by sodium dodecyl sulfate-polyacrylamide gel electrophoresis (SDS-PAGE) on 10% gels and transferred to polyvinylidene difluoride (PVDF) membranes (Millipore). The blots were detected by the primary antibodies against CREB (ABclonal, A11989, 1 : 1000), phospho-CREB (S133) (Abcam, ab32096, 1 : 1000), the PI3K p85 subunit (CST, 4257, 1 : 1000), and Lamin B1 (CST, 13435, 1 : 1000).

### 2.7. Quantitative Real-Time PCR

Total RNA was isolated from the liver and hepatocytes using RNAiso Plus (Takara, Beijing, China) according to the manufacturer's instructions. cDNA was synthesized from 2.0 *μ*g total RNA using the 5x All-In-One RT MasterMix (abm, Richmond, BC), and Real-time PCR (qPCR) was performed using SYBR Green qPCR Master Mix (Bimake, Houston, TX) with an ABI 7900 RT-PCR machine. The primer sets used were as follows: phosphoenolpyruvate carboxykinase (Pepck) forward (5′-TCAGCTGCATAACGGTCTGG-3′) and Pepck reverse (5′-GCTTTCTCAAAGTCCTCTTC-3′), glucose-6-phosphatase (G6pc) forward (5′-GAGGAAGGAATGAACATTCT-3′) and G6pc reverse (5′-GGTCCGGTCTCACAGGTGAC-3′), and 36B4 forward (5′-AAGCGCGTCCTGGCATTGTCT-3′) and 36B4 reverse (5′-CCGCAGGGGCAGCAGTGGT-3′). The expression level of each gene was normalized to the expression of 36B4.

### 2.8. Statistical Analysis

The data are expressed as the mean ± standard error (S.E.). The data between groups were analyzed by ANOVA (GraphPad Prism, La Jolla, CA) or Student's *t*-test. *P* < 0.05 was considered statistically significant.

## 3. Results

### 3.1. Berberine Improves Glucose Metabolism in Diabetic Mice

The leptin-deficient mouse (ob/ob), which is the classic type 2 diabetic model, and the STZ-treated mouse, which is the classic type 1 diabetic model, were used to test the hypoglycemic effect of BBR. Others have previously identified that BBR may interact with the gut microbiota, and this important mechanism may prevent obesity and insulin resistance [21–23]. Therefore, we changed the administration of BBR from gavage to intraperitoneal. The results before the changes are presented in Supplementary Figs. [Supplementary-material supplementary-material-1].

**Figure 1:**
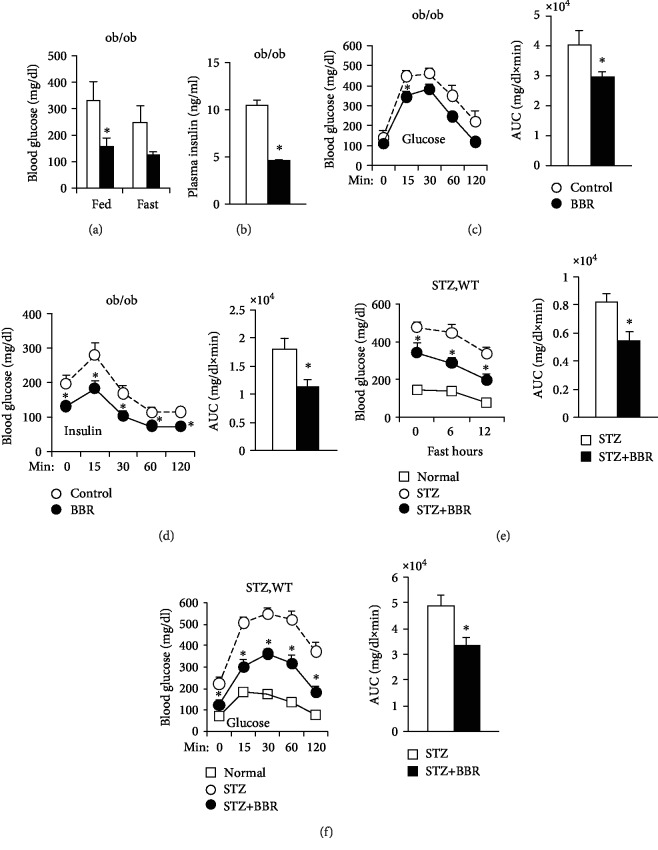
BBR improves glucose metabolism in diabetic mice. (a–d) ob/ob mice were treated with saline (control) or BBR intraperitoneally once a day for 3 weeks while consuming a normal chow diet. (a) Feeding and fasting blood glucose. ^∗^*P* < 0.05, compared with the control group (*N* = 8). (b) Fasting plasma insulin levels in ob/ob mice for 18 weeks. ^∗^*P* < 0.05, compared with the control group (*N* = 8). GTTs (c) and ITTs (d) were performed in ob/ob mice for 19 and 20 weeks, and areas under the curve (AUCs) were calculated. ^∗^*P* < 0.05, compared with the control group (*N* = 8). (e, f) The age-matched mice were treated with saline (normal), and the STZ-induced mice were treated with saline (STZ) or BBR (STZ+BBR) once a day for 3 weeks while consuming a normal chow diet. (e) Fasting blood glucose and 12 hr AUCs of the STZ and BBR groups. ^∗^*P* < 0.05, compared with the STZ group (*N* = 7‐9). (f) Glucose tolerance tests were performed, and areas under the curve (AUCs) were calculated. Each value represents the mean ± S.E.^∗^*P* < 0.05, compared with the STZ group (*N* = 7‐9).

The levels of fed blood glucose and insulin in ob/ob mice were significantly decreased by BBR treatment, which also reduced the fasting blood glucose level but did not reach significant difference (Figures 1(a) and 1(b)). The glucose and insulin tolerance in BBR-treated ob/ob mice was significantly improved compared to those in control mice (Figures 1(c) and 1(d)).

STZ successfully provoked hyperglycemia, and BBR treatment significantly decreased fasting blood glucose (Figure 1(e)) and improved glucose intolerance (Figure 1(f)) in STZ-induced diabetic mice, suggesting that berberine decreased hyperglycemia in an insulin-independent manner.

### 3.2. Berberine Decreased Hepatic Gluconeogenesis in the Livers of Diabetic Mice

**Figure 2:**
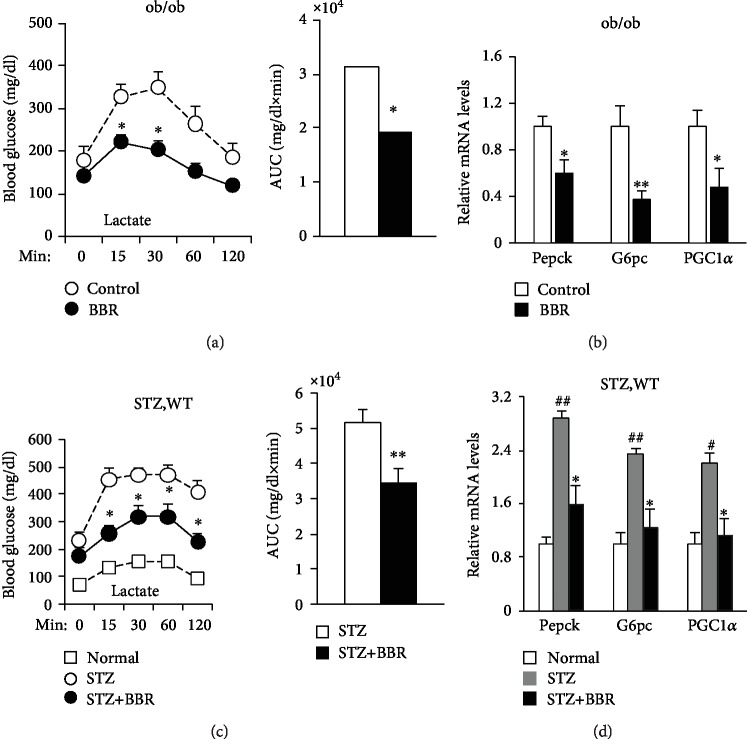
In vivo regulation of the gluconeogenic program by BBR. Lactate tolerance test in diabetic mice. Fasted mice were injected intraperitoneally with either NaCl or sodium lactate. Blood glucose was measured across time and the AUC data. (a) Lactate tolerance test in ob/ob mice. ^∗^*P* < 0.05, compared with the control group (*N* = 8). (c) Lactate tolerance test in STZ-induced diabetic mice. ^∗^*P* < 0.05, ^∗∗^*P* < 0.01, compared with the STZ group (*N* = 7‐9). Quantitative PCR analysis of Pepck, G6pc, and PGC1*α* mRNA levels in livers from diabetic mice and normalized to 36B4 levels. (b) PCR analysis in ob/ob mice. ^∗^*P* < 0.05, ^∗∗^*P* < 0.01, compared with the control group (*N* = 8). (d) PCR analysis in STZ-induced diabetic mice. ^∗^*P* < 0.05, compared with the STZ group; ^#^*P* < 0.05, ^##^*P* < 0.01, compared with the normal group (*N* = 7‐9).

To test the effects of BBR on hepatic gluconeogenesis, we subjected the animals to BBR and lactate, a gluconeogenetic substrate. The hyperglycemia in response to lactate was blunted after BBR treatment in ob/ob (Figure 2(a)) and STZ-induced diabetic mice (Figure 2(c)). Moreover, the mRNA levels of gluconeogenetic genes Pepck, G6pase, and PGC1*α* were downregulated by BBR in the livers of ob/ob (Figure 2(b)) and STZ-induced diabetic mice (Figure 2(d)). These data suggest that BBR reduces hepatic glucose production by downregulating the gluconeogenetic genes.

### 3.3. Berberine Suppressed Glucagon-Induced CREB Phosphorylation and Hepatic Glucose Production

CREB, the main transcriptional factor controlling the expression of gluconeogenetic genes in the liver, is activated by phosphorylation via the glucagon/cAMP/PKA pathway [24]. To examine whether BBR regulates the expression of gluconeogenetic genes by affecting CREB activity, we utilized primary hepatocytes treated with BBR and/or glucagon as the inspection platform.

**Figure 3:**
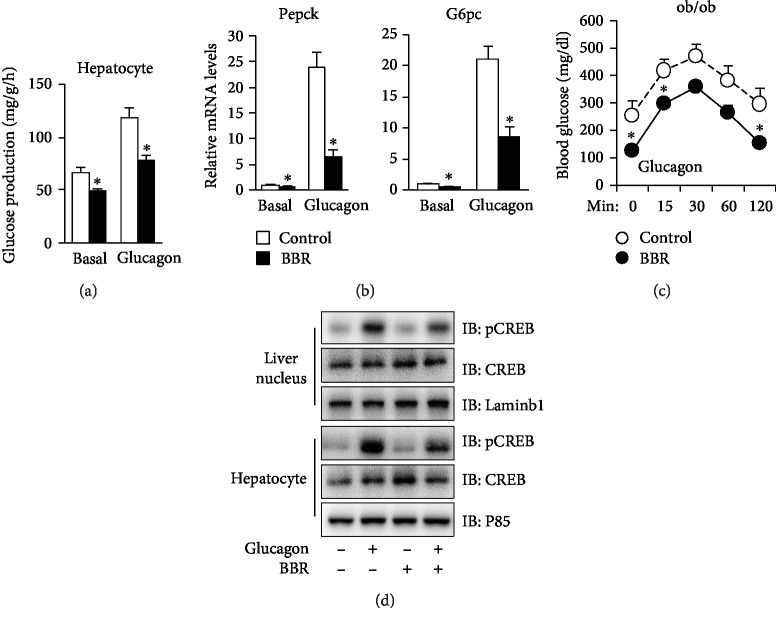
BBR represses glucogenesis in glucagon-induced primary mouse hepatocytes and livers of ob/ob mice. (a) Glucose production from C57BL/6J primary hepatocytes incubated in the absence or presence of 10 *μ*M BBR and/or 50 nM glucagon. ^∗^*P* < 0.05, compared with the control group (*N* = 3). (b) Gene expression measured by quantitative PCR in primary mouse hepatocytes. ^∗^*P* < 0.05, compared with the control group (*N* = 3). (c) Glucagon tolerance test. ^∗^*P* < 0.05, compared with the control group (*N* = 8). (d) CREB protein expression levels in liver homogenates and hepatocytes of ob/ob mice treated with vehicle, glucagon, or BBR, as indicated. The data are expressed as the mean ± S.E. (*N* = 3).

BBR reduced the basal and glucagon-induced glucose production (Figure 3(a)) as well as the mRNA levels of Pepck and G6pase (Figure 3(b)). Furthermore, in ob/ob mice, BBR significantly lowered blood glucose levels stimulated by glucagon (Figure 3(c)), suggesting that BBR inhibits the glucagon signaling pathway. Indeed, we found that BBR inhibited the phosphorylation of CREB in the hepatocytes and livers of ob/ob mice (Figure 3(d)).

### 3.4. Berberine Reversed the Glucagon-Induced Increase in Intercellular cAMP Levels

**Figure 4:**
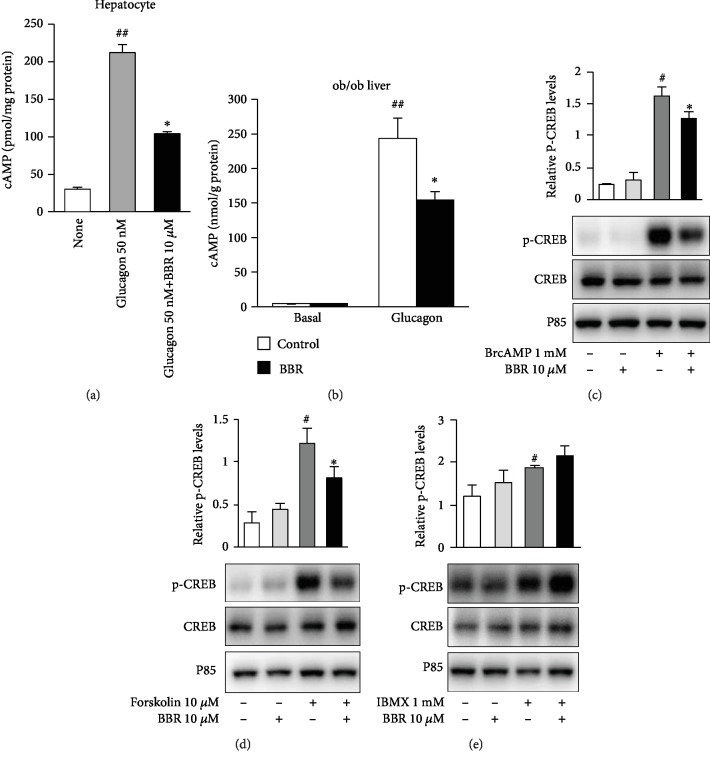
Effects of BBR on cAMP levels and CREB phosphorylation induced by various agonists. (a) cAMP in hepatocytes after glucagon treatment with and without BBR pretreatment. ^##^*P* < 0.01, compared with the none group; ^∗^*P* < 0.05, compared with the glucagon group (*N* = 5). (b) cAMP in the liver with and without intravenous glucagon treatment. ^##^*P* < 0.01, compared with the none group; ^∗^*P* < 0.05, compared with the glucagon group (*N* = 5). (c–e) Primary hepatocytes were preincubated with BBR for 15 min, then stimulated with 1 mM 8-bromo-cAMP (c), 10 *μ*M forskolin (d), or 1 mM IBMX (e) for another 30 min, and protein was analyzed by western blot with the total CREB antibodies and the phospho- (p-) CREB antibody. CREB phosphorylation was normalized to total CREB levels. The data are expressed as the mean ± S.E.^#^*P* < 0.05, compared with the untreated group; ^∗^*P* < 0.05, compared with the agonist group (*N* = 3).

To determine whether BBR inhibits the phosphorylation of CREB via decreasing the cAMP level, we measured cAMP levels in hepatocytes and the liver. The results showed that glucagon dramatically increased cAMP levels in hepatocytes and the liver, and they were significantly reduced by BBR (Figures 4(a) and 4(b)), suggesting that cAMP is involved in the regulation of CREB phosphorylation by BBR. To lock the action target of BBR in the glucagon pathway, we detected the phosphorylation of CREB in response to 8-bromo-cAMP, forskolin, and 3-isobutyl-1-methylxanthine (IBMX). We found that BBR inhibited 8-bromo-cAMP (Figure 4(c)) and forskolin-stimulated (Figure 4(d)) phosphorylation of CREB but could not reverse IBMX-stimulated (Figure 4(e)) phosphorylation, suggesting that BBR may accelerate cAMP degradation by activating PDE.

## 4. Discussion

To relieve hyperglycemia in type 2 diabetes, the predominant therapeutic strategy is to suppress hepatic gluconeogenesis [9, 25]. The present study reveals that BBR attenuates hepatic gluconeogenesis by blocking the glucagon pathway, the main hormone that increases blood glucose.

A variety of studies have demonstrated that BBR attenuates hepatic gluconeogenesis and hyperglycemia by relieving insulin resistance in type 2 diabetes [26–28], which is consistent with the enhanced insulin sensitivity and reduced glucose levels observed in ob/ob mice treated with BBR in the present investigation. However, a hypoglycemic effect caused by BBR was also observed in mice lacking insulin due to the STZ-induced destruction of pancreatic *β*-cells. This suggests that in addition to increasing insulin sensitivity, BBR exerts a hypoglycemic effect in an insulin-independent manner.

The glucagon pathway is suspected of being the target of BBR beyond the insulin pathway because abnormally elevated glucagon levels and increased hepatic glucagon sensitivity drive hepatic gluconeogenesis in type 2 diabetic patients [13]. Indeed, BBR reduced the amount of lactate and glucagon-triggered hepatic glucose production and downregulated gluconeogenetic genes including Pepck, G6pc, and PGC-1*α* in the liver and hepatocytes. CREB, the transcriptional factor regulating the gluconeogenetic genes above, is located downstream of the glucagon signaling pathway. It is activated by phosphorylation induced via the glucagon/glucagon receptor/AC/cAMP/PKA pathway [9, 29]. The level of cAMP, a second messenger transferring signals of glucagon into cells, is controlled by multiple nodes, including cAMP synthetase (AC) and cAMP degrading enzyme (PDE) [30, 31]. We found that BBR inhibited CREB phosphorylation via reducing the level of cAMP in the cytoplasm of hepatocytes. Furthermore, BBR downregulates cAMP by activating PDE. Actually, PDE breaks the phosphodiester bond in cAMP and regulates the localization, duration, and amplitude of cAMP signaling within subcellular domains. PDEs are therefore important regulators of signal transduction mediated by these second messenger molecules.

In conclusion, BBR accelerates intracellular cAMP degradation by activating PDE and thus blocks the hepatic glucagon pathway through downregulating CREB phosphorylation and the downstream gluconeogenic genes, thereby reducing blood glucose in mice. Our data indicate that BBR plays a pivotal role as a regulator of gluconeogenesis in the diabetic state.

## Data Availability

The data used to support the findings of this study are included within the article.

## Supplementary Materials

Supplementary Materials Supplementary Fig. 1: BBR improves glucose metabolism in ob/ob mice. The mice were gavaged with saline (control) or BBR once a day for 3 weeks while consuming a normal chow diet. (A) Feeding and fasting blood glucose. (B) Fasting plasma insulin levels. (C) Glucose tolerance test. (D) Insulin tolerance test. (E) Lactate tolerance test. (F) Glucagon tolerance test. Areas under the curve (AUCs) were calculated. Each value represents the mean ± S.E.^∗^*P* < 0.05, compared with the control group (*N* = 7‐8). Supplementary Fig. 2: BBR improves glucose metabolism in STZ-induced diabetic mice. The mice were gavaged with saline (normal), and the STZ-induced mice were treated with saline (STZ) or BBR (STZ+BBR) once a day for 3 weeks while consuming a normal chow diet. (A) Fasting blood glucose and 12 hr AUCs of the STZ and BBR groups. (B) Glucose tolerance test. (C) Lactate tolerance test. Areas under the curve (AUCs) were calculated. Each value represents the mean ± S.E.^∗^*P* < 0.05, compared with the STZ group (*N* = 7‐8).
