# Supplementary material for: Berberine Attenuates Hyperglycemia by Inhibiting the Hepatic Glucagon Pathway in Diabetic Mice
Source: Oxid Med Cell Longev. 2020 Jan 2;2020:6210526. doi: 10.1155/2020/6210526 (PMC6961611; doi:10.1155/2020/6210526)
Supplement: Supplementary Materials — Supplementary Fig. 1: BBR improves glucose metabolism in ob/ob mice. The mice were gavaged with saline (control) or BBR once a day for 3 weeks while consuming a normal chow diet. (A) Feeding and fasting blood glucose. (B) Fasting plasma insulin levels. (C) Glucose tolerance test. (D) Insulin tolerance test. (E) Lactate tolerance test. (F) Glucagon tolerance test. Areas under the curve (AUCs) were calculated. Each value represents the mean ± S.E.∗P < 0.05, compared with the control group (N = 7‐8). Supplementary Fig. 2: BBR improves glucose metabolism in STZ-induced diabetic mice. The mice were gavaged with saline (normal), and the STZ-induced mice were treated with saline (STZ) or BBR (STZ+BBR) once a day for 3 weeks while consuming a normal chow diet. (A) Fasting blood glucose and 12 hr AUCs of the STZ and BBR groups. (B) Glucose tolerance test. (C) Lactate tolerance test. Areas under the curve (AUCs) were calculated. Each value represents the mean ± S.E.∗P < 0.05, compared with the STZ group (N = 7‐8). [file 6210526.f1.pptx]

## Slide 1
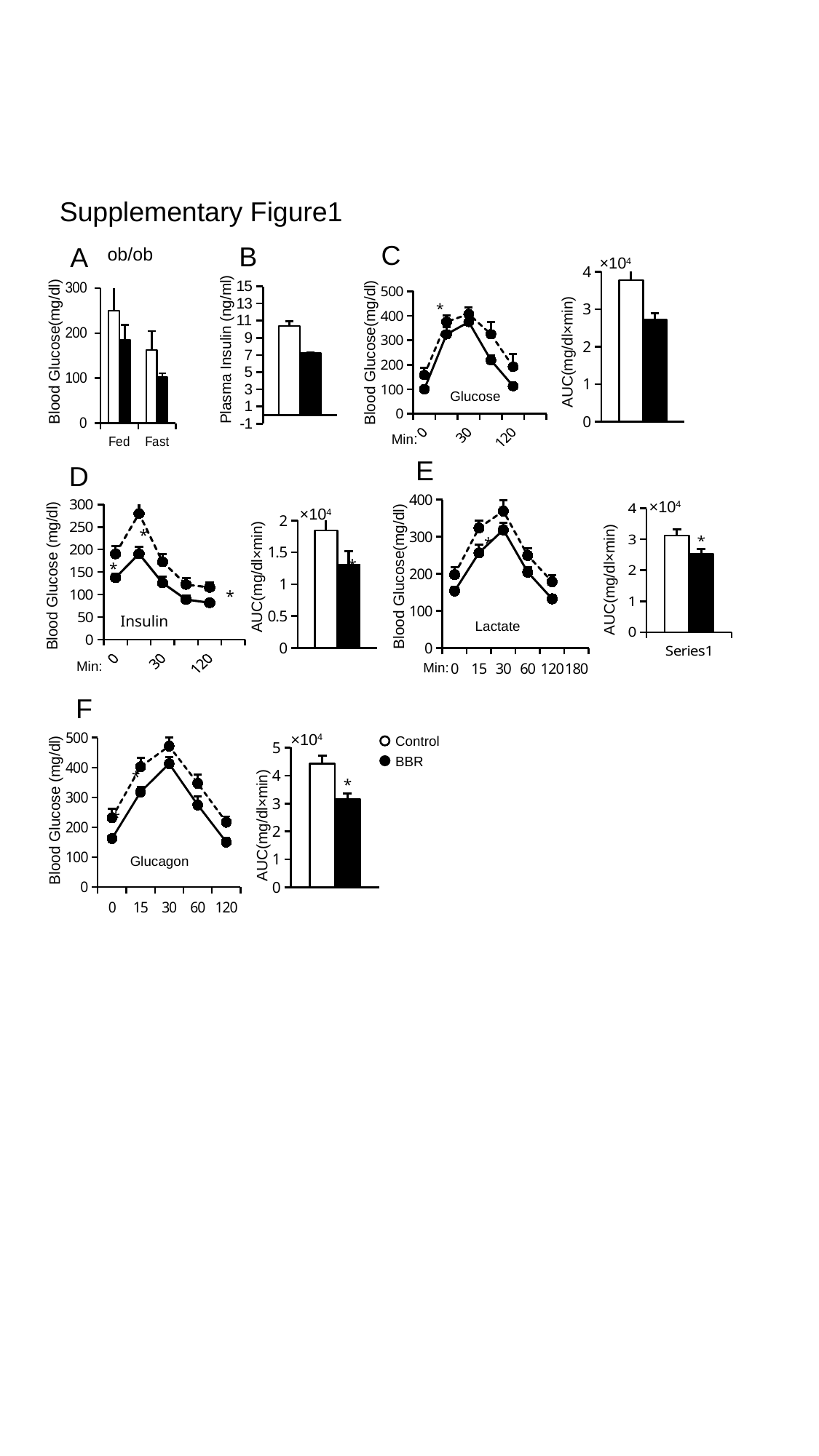

Supplementary Figure1
C
B
A
### Chart
| Category | | |
|---|---|---|
| Fed | 249.58885079161314 | 185.125 |
| Fast | 162.0 | 101.875 |ob/ob
### Chart
| Category | | |
|---|---|---|×104
### Chart
| Category | | |
|---|---|---|
[unsupported chart]
*
Plasma Insulin (ng/ml)
Blood Glucose(mg/dl)
AUC(mg/dl×min)
Blood Glucose(mg/dl)
Glucose
Min:
E
D
[unsupported chart]
[unsupported chart]
×104
### Chart
| Category | | |
|---|---|---|
[unsupported chart]
×104
*
*
*
*
Blood Glucose (mg/dl)
Blood Glucose(mg/dl)
AUC(mg/dl×min)
AUC(mg/dl×min)
*
Insulin
Lactate
Min:
Min:
F
### Chart
| Category | | |
|---|---|---|×104
Control
### Chart
| Category | | |
|---|---|---|
| 0 | 231.9090909090909 | 162.36363636363637 |
| 15 | 402.45454545454544 | 317.72727272727275 |
| 30 | 471.09090909090907 | 412.45454545454544 |
| 60 | 346.90909090909093 | 274.6363636363636 |
| 120 | 216.9090909090909 | 150.45454545454547 |
BBR
*
Blood Glucose (mg/dl)
*
*
AUC(mg/dl×min)
Glucagon

## Slide 2
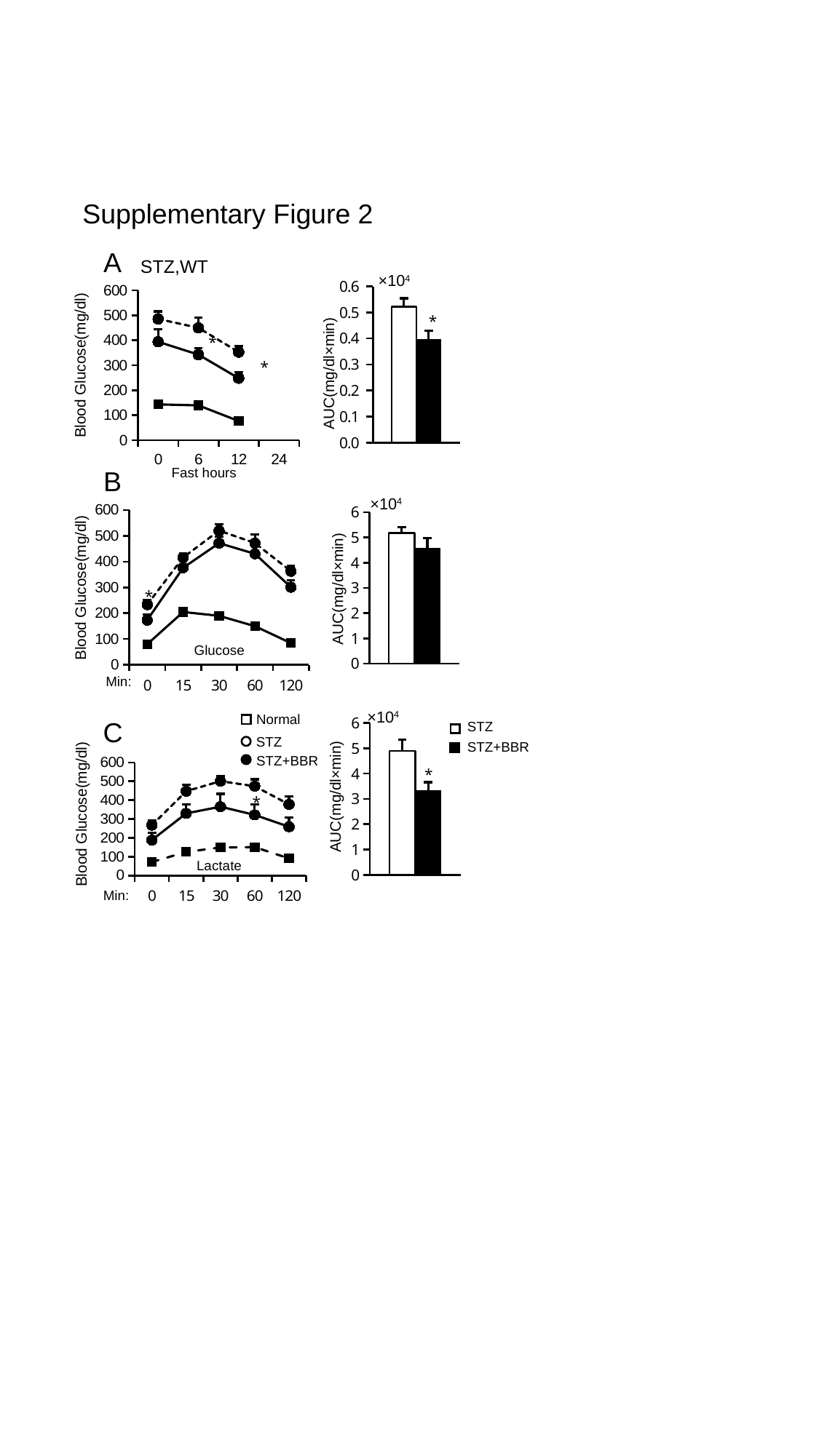

Supplementary Figure 2
A
STZ,WT
### Chart
| Category | STZ | STZ+BBR |
|---|---|---|×104
[unsupported chart]
*
*
Blood Glucose(mg/dl)
*
AUC(mg/dl×min)
B
Fast hours
### Chart
| Category | STZ | STZ+BBR |
|---|---|---|×104
### Chart
| Category | | | |
|---|---|---|---|
| 0 | 79.71428571428571 | 233.22222222222223 | 173.25 |
| 15 | 204.71428571428572 | 415.8888888888889 | 376.0 |
| 30 | 190.0 | 519.8888888888889 | 471.25 |
| 60 | 149.57142857142858 | 471.55555555555554 | 430.0 |
| 120 | 84.28571428571429 | 362.55555555555554 | 300.75 |Blood Glucose(mg/dl)
AUC(mg/dl×min)
*
Glucose
Min:
### Chart
| Category | STZ | STZ+BBR |
|---|---|---|×104
Normal
C
STZ
STZ
STZ+BBR
### Chart
| Category | | | |
|---|---|---|---|
| 0 | 71.57142857142857 | 266.8888888888889 | 188.14285714285714 |
| 15 | 125.28571428571429 | 447.3333333333333 | 329.42857142857144 |
| 30 | 148.85714285714286 | 500.6666666666667 | 365.0 |
| 60 | 150.57142857142858 | 473.8888888888889 | 321.57142857142856 |
| 120 | 91.0 | 377.55555555555554 | 258.7142857142857 |
STZ+BBR
*
AUC(mg/dl×min)
*
Blood Glucose(mg/dl)
Lactate
Min:
